# Hepatoprotective Effect of Ugonin M, A *Helminthostachys* *zeylanica* Constituent, on Acetaminophen-Induced Acute Liver Injury in Mice

**DOI:** 10.3390/molecules23102420

**Published:** 2018-09-21

**Authors:** Kun-Chang Wu, Yu-Ling Ho, Yueh-Hsiung Kuo, Shyh-Shyun Huang, Guan-Jhong Huang, Yuan-Shiun Chang

**Affiliations:** 1 Department of Chinese Pharmaceutical Sciences and Chinese Medicine Resources, College of Chinese Medicine, China Medical University, Taichung 40402, Taiwan; kunchangwu@gmail.com (K.-C.W.); kuoyh@mail.cmu.edu.tw (Y.-H.K.); 2 School of Pharmacy, College of Pharmacy, China Medical University, Taichung 40402, Taiwan; sshuang@mail.cmu.edu.tw; 3 Department of Nursing, Hungkuang University, Taichung 43302, Taiwan; elaine@sunrise.hk.edu.tw; 4 Department of Biotechnology, Asia University, Taichung 41354, Taiwan; 5 Chinese Crude Drug Pharmacy, China Medical University Hospital, Taichung 40402, Taiwan

**Keywords:** acute liver injury, hepatoprotective, acetaminophen, *Helminthostachys zeylanica*, Ugonin M, anti-inflammatory

## Abstract

The present study aimed to discover the possible effectiveness of Ugonin M, a unique flavonoid isolated from *Helminthostachys zeylanica*—a traditional Chinese medicine used as anti-inflammatory medicine—and to elucidate the potential mechanisms of Ugonin M in the acute liver injury induced by acetaminophen (APAP). In this study, Ugonin M significantly ameliorated APAP-induced histopathological changes and the typical liver function biomarkers (i.e., alanine aminotransferase (ALT), aspartate aminotransferase (AST), and total bilirubin (T-Bil)). It also affected APAP-induced abnormal lipid metabolism including total cholesterol (TC) and triglyceride (TG) in the serum. In inflammatory pharmacological action, Ugonin M suppressed the pro-inflammatory mediators such as nitric oxide (NO) and the lipid peroxidation indicator malondialdehyde (MDA). In addition, Ugonin M reinforced hemeoxygenase-1 (HO-1) protein expression and the production of antioxidant enzymes viz superoxide dismutase (SOD), glutathione peroxidase (GPx), and catalase (CAT). Furthermore, inflammation-associated cytokines including tumor necrosis factor-*α* (TNF-*α*), interleukin-6 (IL-6), and IL-1*β* as well as proteins such as inducible nitric oxide synthase (iNOS) and cyclooxygenase-2 (COX-2) were decreased by the pretreatment of Ugonin M. Moreover, this study found that pretreatment of Ugonin M apparently decreased nuclear factor-kappa B (NF-*κ*B) and mitogen-activated protein kinases (MAPKs) activation via inhibition of the degradation of NF-*κ*B, inhibitory κB-*α* (I*κ*B-*α*)*,* extracellular regulated kinase (ERK), c-Jun-*N*-terminal (JNK), and p38 active phosphorylation. In conclusion, Ugonin M significantly showed a protective effect against APAP-induced liver injury by reducing oxidative stress and inflammation. Thus, Ugonin M could be one of the effective components of *H. zeylanica* that plays a major role in the treatment of inflammatory disorders.

## 1. Introduction

Acetaminophen (APAP), known as a popular analgesic and antipyretic agent, has been a highly utilized over-the-counter medication worldwide for decades. It is generally effective and safe to be used within the therapeutic doses. However, it is one of the commonest causes of acute liver injury due to the occurrence of overdose or abuse and its dose-related hepatocellular necrosis by APAP [1,2].

Normally, the APAP is metabolized to non-toxic metabolites by glucuronides or sulfates in hepatocytes before it is finally excreted in the urine. However, once the capacity of normal metabolic pathways is saturated, surplus APAP will be metabolized by the cytochrome P450 (CYP) enzymes (most notably CYP2E1), producing a highly reactive and toxic intermediate *N*-acetyl-*p*-benzoquinone imine (NAPQI) and reactive oxygen species (ROS) [3,4]. Glutathione (GSH) could immediately metabolize and neutralize NAPQI to harmless mercapturic acid. However, once GSH is depleted, the remnant NAPQI may cause hepatocyte damage via the increased formation of mitochondrial protein adducts and the subsequent oxidative stress caused by superoxide radicals and peroxynitrite as well as lipid peroxidation [5,6,7]. Moreover, the activation of the mitogen activated protein kinase (MAPK), c-Jun-*N*-terminal kinase (JNK), and the subsequent inflammatory responses result in the programmed injury [6,8]. It is evidenced that both oxidative stress [4,6,8,9,10] and inflammatory response [2,6,10] are involved in hepatic injury in APAP-induced liver injury model.

At present, *N*-acetylcysteine (NAC) is the only antidote that is approved and indicated in clinical practice for dose-dependent APAP-induced hepatotoxicity; it works by increasing the synthesis of glutathione and subsequently detoxifying NAPQI to non-toxic intermediate mercapturic acid [5,11].

*Helminthostachys zeylanica* (L.) Hook. (HZ) is a terrestrial, herbaceous fern ally in the Ophioglossaceae family, and the roots and rhizomes of HZ, known colloquially as “Ding-Di-U-Gon”, have been traditionally used in a variety of treatments such as of inflammation, burns, fever, and pneumonia [12,13]. Current studies used the crude extracts of HZ to evaluate the anti-inflammatory effects through in vivo hepatotoxicity [14,15] and acute lung injury models [16]. Several unique flavonoids isolated from HZ were reported to possess a variety of biological activities including antioxidant activities [17,18], anti-inflammatory activities [17,19,20,21,22,23,24], melanogenesis inhibitory activities [25,26]; neuroprotection [27], antiosteoporosis [23,28,29], anti-cancer [30,31], and immunomodulatory effects [32]; and prevention of neointimal hyperplasia and migration [33].

**Figure 1:**
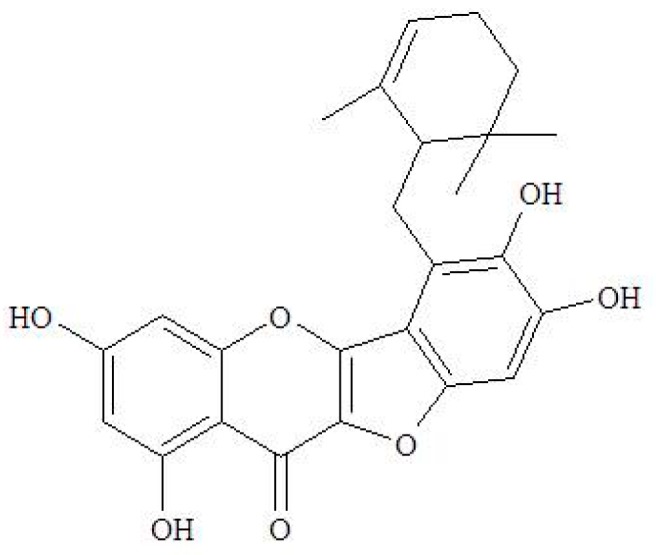
Structure of Ugonin M.

Ugonin M (Figure 1), a unique flavonoid, can currently only be isolated from HZ. Huang YC et al. showed an in vitro anti-inflammatory activity of Ugonin M [19]. Our previous study showed through HPLC analysis that Ugonin M is one of the major representative components of HZ [34] and subsequently it was found to also present some protection effect from acute lung injury induced by lipopolysaccharides in the in vivo study [24]. The results of the previous effort aligned with the study from Suja SR et al. whereby it showed that the crude extract of HZ possesses hepatoprotective effects against harmful substance [14,15]. However, the effective component of HZ in the pharmacological effect of hepatoprotection and further mechanisms of Ugonin M in APAP-induced liver injury are still ambiguous and are yet to be discovered. Thus, the present research mainly aimed to investigate the involvement of Ugonin M in anti-inflammatory action and to discover its potential mechanism of hepatoprotective action through an APAP-induced liver injury model.

## 2. Results

### 2.1. Effects of Ugonin M on APAP-Induced Liver Injury

**Figure 2:**
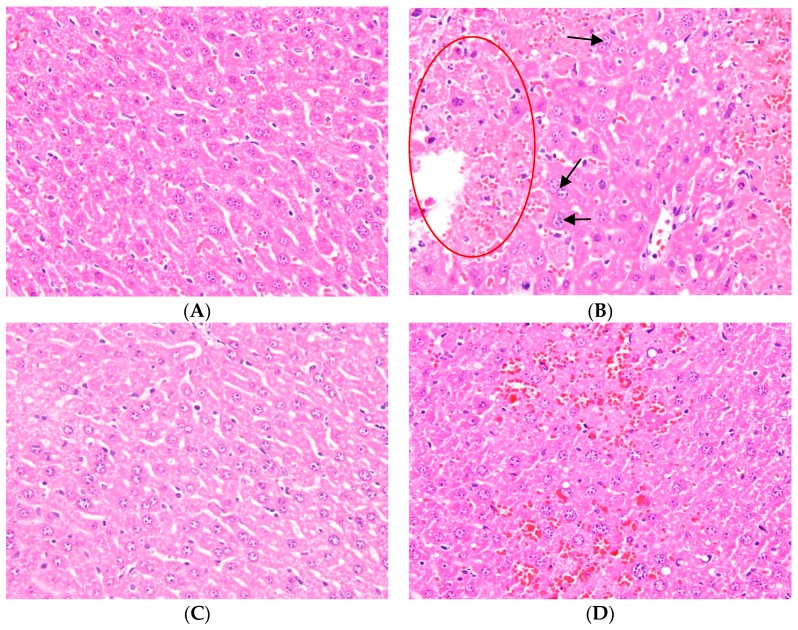

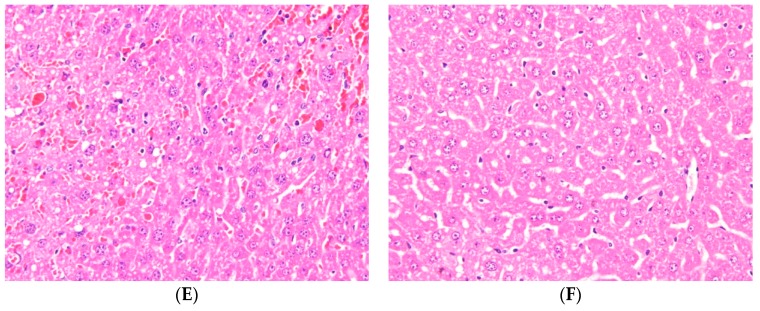
Ugonin M pretreatment alleviated acetaminophen (APAP)-induced liver injury in mice. (**A**) Control group; (**B**) APAP-only group: presence of severe centrilobular necrosis in the defined area (red oval), polymorphonuclear inflammatory infiltrates (black arrows), and vacuolated hepatocytes; (**C**) APAP + 600 mg/kg *N*-acetylcysteine (NAC) group; (**D**) APAP + 0.625 mg/kg Ugonin M group; (**E**) APAP + 1.25 mg/kg Ugonin M group; (**F**) APAP + 2.5 mg/kg Ugonin M group. The figures demonstrate representative views of hematoxylin and eosin (H&E) stained liver tissue from each group. The original magnification: ×400.

Hematoxylin and eosin (H&E) staining of liver tissues was used in the present study for the observation of pathological changes. The results in Figure 2B show that typical pathological changes of APAP-induced hepatotocixity resulted in an increased vacuolization and centrilobular necrosis when compared with the control group (Figure 2A). Figure 2C shows the reduction of pathological changes after the pretreatment of NAC. Three different doses of Ugonin M (0.625 mg/kg, 1.25 mg/kg, and 2.5 mg/kg) showed improvement (reduction) of the vacuolization and centrilobular necrosis (Figure 2D–F). Interestingly, it can be seen that at 2.5 mg/kg, the H&E staining of liver was similar to both the control group and the group with pretreatment of NAC. Hence, the results showed that there are significant histopathological changes between groups and it can be concluded that the pretreatment of Ugonim M (dose dependent) has a protective effect towards APAP-induced injured liver tissue.

### 2.2. Effects of Ugonin M on Liver Functions

**Figure 3:**
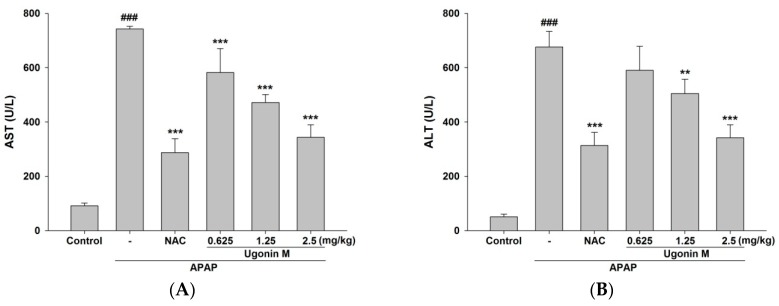

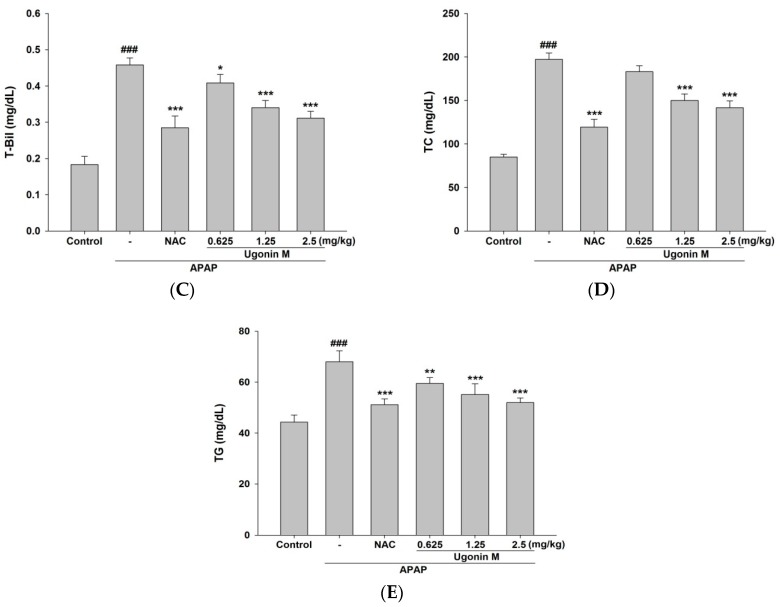
Effects of Ugonin M on serum (**A**) aspartate aminotransferase (AST), (**B**) alanine aminotransferase (ALT), (**C**) total bilirubin (T-Bil), (**D**) total cholesterol (TC), and (**E**) triglyceride (TG). Data are expressed as mean M ± SD; *n* = 6.

APAP-induced acute liver injury caused elevation of serum aspartate aminotransferase (AST), alanine aminotransferase (ALT), and total bilirubin (T-Bil) levels in mice treated with APAP but not in the control group (Figure 3A–C). However, because of the pretreatment of Ugonin M and NAC, the elevation of serum AST, ALT, and T-Bil levels were significantly reduced especially through pretreatment of Ugonin M (1.25 and 2.5 mg/kg) and NAC (600 mg/kg) compared to the APAP-only group. As for a lower dose of Ugonin M (0.625 mg/kg), there were significant reductionsin serum AST (*p* < 0.001) and T-Bil (*p* < 0.05) levels but not in the ALT level. The results shown in Figure 3A–C demonstrate that Ugonin M held some dose dependent protective abilities against APAP-induced liver injury. Besides, the pretreatment of Ugonin M (1.25 and 2.5 mg/kg) and NAC (600 mg/kg) also significantly reduced the elevated serum triglyceride (TG) and total cholesterol (TC) levels compared with the APAP group (*p* < 0.001) (Figure 3D,E). Again, at a lower dose of Ugonin M (0.625 mg/kg), there was a significant change in TG but not TC levels.

### 2.3. Effects of Ugonin M on Lipid Peroxidation in Liver Tissue

**Figure 4:**
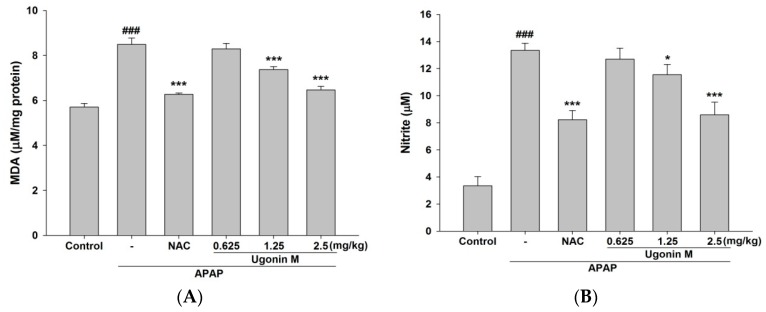
Effects of Ugonin M on (**A**) liver peroxide level and (**B**) serum nitric oxide (NO) level. Data are expressed as M ± SD; *n* = 6.

In the malondialdehyde (MDA) assay, the MDA level was obviously increased in the APAP-only group compared with the control group. The pretreatment of Ugonin M (1.25 and 2.5 mg/kg) and NAC (600 mg/kg) significantly inhibited the increase of the lipid peroxidation marker when compared with that of the APAP-only group (*p* < 0.001) (Figure 4A).

### 2.4. Effects of Ugonin M on Serum Nitric Oxide (NO) Levels

The NO level was obviously increased in the APAP-only group compared with that of the control group. The pretreatment of Ugonin M (1.25 and 2.5 mg/kg) and NAC (600 mg/kg) significantly reduced the increase in NO level compared with that of the APAP-only group (*p* < 0.05 and *p* < 0.001, respectively) (Figure 4B).

### 2.5. Effects of Ugonin M on the Activity of Antioxidant Enzymes in Liver Tissue

**Figure 5:**
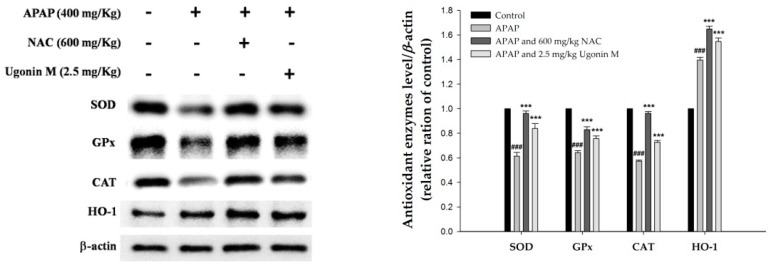
The effects of Ugonin M towards antioxidant enzymes expression (superoxide dismutase (SOD), glutathione peroxidase (GPx), catalase (CAT), and hemeoxygenase-1 (HO-1)) in liver tissues. Data are expressed as M ± SD; *n* = 3.

In order to further determine the antioxidant ability of Ugonin M, the activities of antioxidant enzymes of superoxide dismutase (SOD), glutathione peroxidase (GPx), and catalase (CAT) as well as hemeoxygenase-1 (HO-1) in the liver tissue were also measured in this study. As shown in Figure 5, the expression of SOD, GPx, CAT, and HO-1 in the groups pretreated with Ugonin M (2.5 mg/kg) and NAC (600 mg/kg) were significantly higher than that in the APAP-only group (*p* < 0.001). These data demonstrate that Ugonin M may increase the expression of antioxidant enzymes and subsequently decrease the oxidative burden induced by APAP.

### 2.6. Effects of Ugonin M on Serum tumor necrosis factor-α (TNF-α), interleukin (IL)-6, and IL-1β Levels

**Figure 6:**
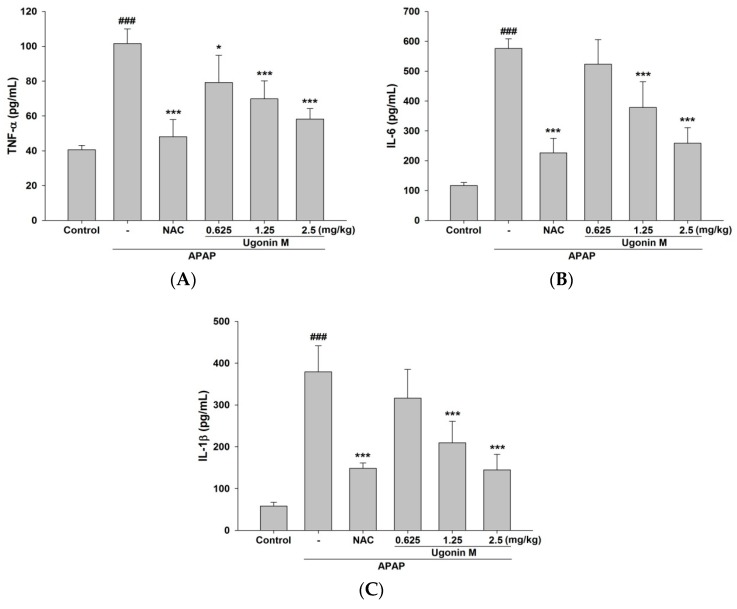
Effects of Ugonin M on serum (**A**) tumor necrosis factor-*α* (TNF-*α)*, (**B**) interleukin (IL)-6, and (**C**) IL-1*β*. Data are expressed as M ± SD; *n* = 6.

As shown in Figure 6, the pro-inflammatory cytokines of TNF-*α*, IL-6, and IL-1*β* were markedly increased in the APAP-only group compared with that of the control group. The levels of TNF-*α*, IL-6, and IL-1*β* in the pretreatment of Ugonin M (1.25 and 2.5 mg/kg) and NAC (600 mg/kg) groups decreased significantly (*p* < 0.001) (Figure 6). The results shown in Figure 6 demonstrate that Ugonin M possesses in vivo anti-inflammatory activity and subsequently decreases the secretion of the pro-inflammatory cytokines including TNF-*α*, IL-6, and IL-1*β* induced by APAP.

### 2.7. Effects of Ugonin M on Inducible Nitric Oxide Synthase (iNOS) and Cyclooxygenase-2 (COX-2) Protein Expression in Liver Tissue

**Figure 7:**
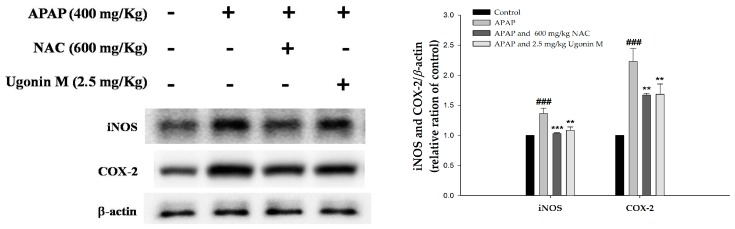
Effects of Ugonin M on inducible nitric oxide synthase (iNOS) and cyclooxygenase-2 (COX-2) protein expression in liver tissue. Data are expressed as M ± SD; *n* = 3.

This research also investigated the level of cytokine proteins in the APAP-induced liver tissues. As shown in Figure 7, the expression of the iNOS and COX-2 proteins in the Ugonin M (2.5 mg/kg) and NAC (600 mg/kg) pretreated groups were significantly inhibited compared to those of the APAP-only group.

### 2.8. Effects of Ugonin M on Activities of Nuclear Factor-Kappa B (NF-κB) in Liver Tissue

**Figure 8:**
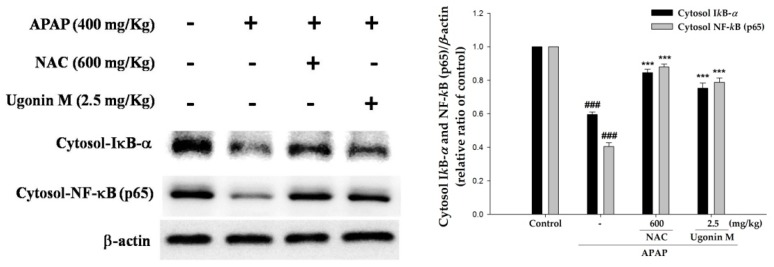
Effects of Ugonin M on APAP-induced cytosolic inhibitory κB-*α* (I*κ*B-*α*) and nuclear factor-kappa B (NF-*κ*B) expression in liver tissue. Data are expressed as M ± SD; *n* = 3.

To understand the effect of Ugonin M on the degradation of inhibitory κB-*α* (I*κ*B-*α*) and the nuclear translocation of NF-*κ*B, the cytosolic fraction of I*κ*B-*α* and NF-*κ*B were evaluated in this study. As shown in Figure 8, it is obvious that the degradation of I*κ*B-*α* and the translocation of NF-*κ*B p65 increased significantly in the APAP-only group compared with that of the control group. The pretreatment of Ugonin M (2.5 mg/kg) and NAC (600 mg/kg) significantly inhibited the degradation of I*κ*B-*α* and the translocation of NF-*κ*B induced by APAP (*p* < 0.001). Hence, the data suggest that Ugonin M prevented APAP-induced liver damage through activation of NF-*κ*B.

### 2.9. Effects of Ugonin M on Activities of MAPK in Liver Tissue

**Figure 9:**
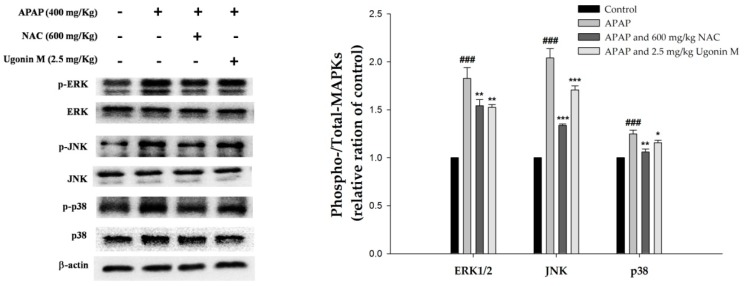
Effects of Ugonin M on APAP-induced mitogen-activated protein kinase (MAPK) phosphorylation and non-phosphorylation protein expression concentration in liver tissue. Data are expressed as M ± SD; *n* = 3.

The MAPK pathway consists of three routes viz extracellular regulated kinase (ERK), JNK, and p38MAPK. It is reported that the MAPK pathway is involved in the expression of NF-*κ*B in the nuclei and subsequent cytokine proteins such as iNOS and COX-2 as well as the pro-inflammatory cytokines such as TNF-*α*, IL-6, and IL-1*β* [35]. Thus, the effect of Ugonin M on the phosphorylation of ERK, JNK, and p38MAPK was further evaluated. As shown in Figure 9, the phosphorylation of ERK, JNK, and p38MAPK were obviously increased in the APAP-only group compared with those the control group. Because of the pretreatment of Ugonin M (2.5 mg/kg) and NAC (600 mg/kg), the phosphorylation of ERK, JNK, and p38MAPK all significantly decreased compared with those of the APAP-only group. The results shown in Figure 6, Figure 7, Figure 8 and Figure 9 indicate that one of the mechanisms of Ugonin M ameliorating APAP-induced liver injury was similar to NAC (positive control), which was through the inactivation of NF-*κ*B and MAPK as well as subsequent pro-inflammatory cytokines.

## 3. Discussion

The crude extract of HZ demonstrated hepatoprotective effects in the traditional use and was proven in previous reports [14,15]. Phytochemical research has reported that HZ is abundant in flavonoids [18,19,20,22,23]. Ugonin M, one of the major flavonoid components of HZ [34], exhibited both in vitro [19,23] and in vivo [24] antioxidant and anti-inflammatory activities. Thus, this study aimed to elucidate the role of Ugonin M in the pharmacological actions of HZ and subsequently to further discover the mechanism of actions of hepatoprotection viz APAP-induced acute liver injury in animal models [36].

The featured lesion of APAP-induced liver injury is centrilobular hepatocellular necrosis, polymorphonuclear inflammatory infiltrates, and hepatocyte vacuolization [5,37]. Histopathological examination clearly showed that vacuolization and centrilobular necrosis in APAP-induced mice were attenuated by the pretreatment of Ugonin M (Figure 2). The serum hepatic biomarkers of AST, ALT, and T-Bil are classical, sensitive indicators of early acute liver damage, which is linked to oxidant stress [36,38]. This study showed that Ugonin M pretreatment markedly reduced the elevation of the serum AST, ALT, and T-Bil induced by APAP (Figure 3A–C). APAP-induced liver dysfunction also caused abnormal lipid metabolism in TC and TG (Figure 3D,E). Hence, the study presented that Ugonin M may revert the APAP-induced serum AST, ALT, T-Bil, TC, and TG levels. Moreover, the histopathological changes of the liver indicated that there was a direct hepatoprotective effect in APAP-induced acute liver injury.

Studies reported that excessive oxidative stress would cause deleterious processes in the APAP-induced liver injury [37,39,40]. It is emphasized that scavenging free radicals are useful for ameliorating liver injury [41]. As MDA is widely used as an indicator of oxidative stress through oxidative degradation of polyunsaturated fatty acids, it was used as a marker in this study [37]. The free radical NO is a highly reactive oxidant originated from l-arginine through NO synthase, which is increased by the overdose of APAP. NO may react with various ROS to form peroxynitrite, which causes a cytotoxic effect on neutrophils and aggravates lipid peroxidation [42]. The results of this study showed that Ugonin M inhibited the formation of MDA, NO, and iNOS (Figure 4 and Figure 7). Thus, Ugonin M could effectively reduce the impairment of free radicals and lipid peroxidation induced by APAP.

HO-1 serves as a rate-limiting enzyme that catalyzes heme to antioxidant and anti-inflammatory substances viz biliverdin, carbon monoxide, and iron and subsequently ameliorates symptoms of APAP-induced liver injury [35,43]. The expression of HO-1 will rapidly be up-regulated by oxidative stress conditions such as the administration of APAP [44]. However, comparing between the group of pretreatment with Ugonin M and the APAP-only group, a significant increment in HO-1 expression indicated that Ugonin M exerts the counteracting effect by enhancing the expression of HO-1 beyond the normal cellular stress response against APAP-induced oxidative stress. A previous study showed that the severity of APAP-induced liver injury was also modulated by antioxidant enzymes such as SOD, GPx, and CAT [37]. This study found that the pretreatment of Ugonin M may increase the production of SOD, GPx, CAT, and HO-1 (Figure 5). The decreased levels of MDA and NO, and the increased levels of SOD, GPx, CAT, and HO-1 suggest that Ugonin M possesses a hepatoprotective effect through the equilibrium process of APAP-induced oxidative stress.

APAP toxicity modulated by the complex network of inflammatory cells and cytokines, such as TNF-*α* and other pro-inflammatory cytokines, has been studied for decades [37]. Previous articles have proven the process of APAP-induced activation of Kupffer cells, through the increased levels of both pro-inflammatory and anti-inflammatory cytokines [45]. Moreover, TNF-*α* is linked to the increase of oxidative stress by forming the recruited signaling molecule complex I (binding of TNF-*α* to TNF-receptor 1 (TNF-R1)) and subsequently could recruit and activate other inflammatory cells, especially when it activates two central JNK and NF-*κ*B pathways [45]. Further, a previous report showed that COX-2, but not COX-1, was induced in livers of APAP-treated mice. There was an association of oxidative stress and inflammation in APAP-induced hepatotoxicity [35,46]. The results of this study discovered that Ugonin M could down-regulate the level of COX-2 and pro-inflammatory cytokines including TNF-*α*, IL-6, and IL-1*β*, indicating that Ugonin M could mitigate the inflammatory response induced by APAP (Figure 6 and Figure 7).

Since both synthesis of pro-inflammatory cytokines (such as TNF-*α*, IL-6, and IL-1*β*) and the expression of iNOS and COX-2 were regulated by NF-*κ*B, it can be concluded that NF-*κ*B plays a crucial role in the expression of pro-inflammatory genes [47,48]. In an unstimulated situation, NF-*κ*B is normally bound with I*κ*B-*α* and I*κ*B-*β* and is found in cytosol. Once they are subjected to stimulation, I*κ*B-*α* kinase (IKK-*α*) phosphorylates I*κ*B-*α*, causing the release of NF-*κ*B from I*κ*B. The unbound NF-*κ*B would translocate into the nucleus and result in the transcription of most pro-inflammatory cytokines including TNF-*α*, IL-6, and IL-1*β* as well as iNOS and COX-2 [24,47,48].

Cellular MAPK family proteins, including three major pathways viz ERK, p38MAPK, and JNK, take part in a number of cellular functions such as cell death and survival, proliferation, migration, oxidative stress, and inflammatory response to APAP-induced hepatotoxicity [49,50,51,52]. Moreover, several studies have proposed the mechanisms of NF-*κ*B activation that could control the level of JNK activation [22]. Thus, the activity of MAPKs, the degradation of cytosolic fraction of I*κ*B-*α,* and nuclear translocation of NF-*κ*B were examined to evaluate the potential of Ugonin M. The key result from this study (Figure 8) showed that pretreatment with Ugonin M significantly prevented APAP-induced degradation of I*κ*B-*α* and the nuclear translocation of NF-*κ*B. Further, this study also found that APAP stimulation obviously increased MAPK phosphorylation, and Ugonin M significantly suppressed phosphorylation of ERK, JNK, and p38MAPK cascades induced by APAP (Figure 9). Those results indicate that Ugonin M possesses a protective ability against APAP-induced liver injury by inhibiting the NF-*κ*B and MAPK signaling pathways. The findings (Figure 8 and Figure 9) were consistent with the results of the oxidative stress (Figure 4 and Figure 5) and inflammation (Figure 6 and Figure 7), which were associated with protein expressions and enzyme markers.

Ugonin M, the major component of HZ, exhibited hepatoprotective effects against APAP-induced hepatotoxicity via its antioxidant and anti-inflammatory activities. The mechanism of the hepatoprotective effect of Ugonin M was evident through histopathological evaluations of liver tissues, the reduction level of NF-*κ*B and MAPK mediated signaling pathways, decreased levels of the liver function biomarkers and lipid peroxidation indicators, for example MDA, and the increase of the production/expression of antioxidant proteins including SOD, GPx, CAT, and HO-1. Based on our findings in this study, it can be concluded that Ugonin M may be preliminarily proved as one the effective components in the hepatoprotection action in HZ.

## 4. Materials and Methods

### 4.1. The Source of Ugonin M

The raw material of Ugonin M was isolated from HZ that was authenticated and deposited in our laboratory. The structure of Ugonin M was identified by detailed analysis of 1D-NMR spectroscopic data (Bruker DRX-500 FT-NMR, Bruker, Bremen, Germany), and the result was confirmed by comparison with previous published literature studies [19]. All the isolation protocols and spectroscopic data were reported in our previous publication [24].

### 4.2. Animal and Treatments

Experiments were performed on Bltw: CD1 (ICR) male mice, 6 weeks old, that were obtained from BioLASCO Co., Ltd. (Taipei, Taiwan). The animals were kept in plexiglass cages at a constant temperature of 22 ± 1 °C, relative humidity 55 ± 5%, and with 12 h dark-light cycles. They were given food and water *ad libitum*. The animal studies were conducted according to the regulations of the Instituted Animal Ethics Committee, and the animal use protocol (Protocol No.: 2017-228-1; date of approval: 2017.05.17) was approved by the Institutional Animal Care and Use Committee, China Medical University. After an adaptation period of seven days, male ICR mice were randomly divided into the following six groups (*n* = 6): (1) control group, (2) APAP-only group (negative control), (3) APAP + NAC group (positive control), (4) APAP + Ugonin M (0.625 mg/kg) group, (5) APAP + Ugonin M (1.25 mg/kg) group, and (6) APAP + Ugonin M (2.5 mg/kg) group. In the treatment groups, the mice were pretreated by intraperitoneal (*i.p.*) injection of Ugonin M in different concentrations *(*i.e., 0.625, 1.25, and 2.5 mg/kg in 1% carboxymethylcellulose) and NAC (600 mg/kg in phosphate buffered saline (PBS)) once daily for six consecutive days. The mice in the control and APAP-only groups received PBS only. One hour after the final treatment, the acute liver injury was induced by an *i.p.* injection of APAP (400 mg/kg) in all groups other than the control group. APAP was made immediately prior to its use in warm PBS (pH 7.4). The mice were starved for 12 h after the APAP treatment and subsequently euthanized. Blood samples were collected from the carotid arteries for analysis. APAP and NAC were purchased from Sigma-Aldrich Inc. (St. Louis, MO, USA). Throughout the study period, none of the mice were excluded as there were no death cases reported.

### 4.3. Histological Examination

The anterior portion of the left lateral liver lobe from each mouse was fixed in 10% formaldehyde phosphate buffer, embedded in paraffin, cut into 5 μm sections, and then treated with hematoxylin and eosin (H&E) stain for histological examination under light microscopy (Nikon, ECLIPSE, TS100, Tokyo, Japan). Images were captured with a digital camera (KODAK GEL Logic 1500 Camera, Carestream Health, Inc., New York, NY, USA) at an original magnification of 400×.

### 4.4. Assessment of Liver Functions

To obtain the serum, the blood samples were centrifuged at 1700× *g* (Beckman GS-6R, Krefeld, Germany) for 30 min at 4 °C. The biochemical parameters of alanine aminotransferase (ALT), aspartate aminotransferase (AST), total bilirubin (T-Bil), total cholesterol (TC), and triglyceride (TG) were analyzed using clinical test kits (HUMAN Diagnostics Worldwide, Magdeburg, Germany) with a chemical analyzer (Roche Diagnostics, Cobas Mira Plus, Rotkreuz, Switzerland), according to the manufacturer’s instructions.

### 4.5. The Measurement of Nitric Oxide and MDA

The nitrite level, which reflects intracellular nitric oxide (NO) synthase activity, was tested based on the Griess reaction. One hundred μL of Griess reagent (1% sulfanilamide, 0.1% naphthyl ethylenediamine dihydrochloride, and 5% phosphoric acid) was added to each sample and incubated at room temperature for 10 min. Absorbance was recorded at 540 nm. Nitrite levels in the serum samples were obtained by calculation from a standard curve of sodium nitrite [53]. The malondialdehyde (MDA) levels in the liver tissue were determined with the thiobarbituric acid reacting substance (TBARS) method. Briefly, MDA reacted with thiobarbituric acid under an acidic condition at a high temperature and formed a red-complex TBARS. Absorbance was read at 535 nm [41].

### 4.6. TNF-α, IL-6, and IL-1β Cytokines in Serum

The serum concentration of the pro-inflammatory cytokines (i.e., tumor necrosis factor-*α* (TNF-*α*), interleukin-6 (IL-6), and IL-1*β*) in serum were assessed with relevant enzyme-linked immunosorbent assay (ELISA) kits (Biosource International Inc., Sunnyvale, CA, USA) based on the manufacturer’s instructions.

### 4.7. Western Blot Analysis of the Liver Tissues

Lysis buffer, composed of 0.6% NP-40, 150 mM NaCl, 10 mM HEPES (pH 7.9), 1 mM EDTA, and 0.5 mM PMSF, was used in the homogenization of liver tissues at 4 °C. The homogenized samples were then centrifuged at 3000 revolutions per minute (rpm) at 4 °C for 10min to obtain the supernatant. The total cellular protein amounts of supernatant were determined by the protein standard of bovine serum albumin (BSA). Protein samples (50 µg) were resolved by denaturing 10% sodium dodecyl sulfate-polyacrylamide gel electrophoresis (SDS-PAGE) using standard methods, and then were transferred onto PVDF membranes (Immobilon, Millipore, Bedford, MA, USA) for electroblotting and blocking with 10% skim milk. The membranes were incubated with an appropriate dilution of specific primary antibodies (i.e., superoxide dismutase (SOD), glutathione peroxidase (GPx), catalase (CAT), hemeoxygenase-1 (HO-1), inducible nitric oxide synthase (iNOS), cyclooxygenase-2 (COX-2), cytosolic nuclear factor-kappa B (NF-*κ*B), and cytosolic I*κ*B-*β* phosphorylated and non-phosphorylated forms of extracellular regulated kinase 1/2 (ERK1/2), p38 mitogen-activated protein kinase (MAPK), c-Jun-*N*-terminal kinase (JNK) at 4 °C, washed three times with *tris*-buffered saline containing 0.1% tween-20 (TBST), and subsequently incubated for 1 h at 37 °C with horseradish peroxidase-conjugated secondary antibodies (overnight). The membranes were washed three times before examination for immuno-reactive proteins by enhanced chemiluminescence (ECL) reagent (Thermo Scientific, Hudson, NH, USA). Band intensity on scanned films were quantified and represented as relative intensity by comparing with the control group using Image J Software (NIH, Bethesda, MD, USA).

### 4.8. Statistical Analysis

The data obtained from animal experiments were reported as the mean values ± standard deviation (M ± SD), and statistical comparisons between the groups were carried out by one-way ANOVA, followed by a Scheffe’s multiple range test. The criterion for statistical significance was set at a *p* value of less than 0.05. The * sign indicates significant value for comparison with the APAP-only group while # indicates significant value for comparison with the control group; whereby the level of significance were plotted by * *p* < 0.05, ** *p* < 0.01, and *** *p* < 0.001.

## 5. Conclusions

Ugonin M not only effectively attenuated the production of pro-inflammatory mediators as well as the typical liver function biomarkers and abnormal lipid metabolism, but also ameliorated the severity of liver impairment induced by APAP. Moreover, Ugonin M significantly suppressed APAP-induced oxidative stress. In addition, the in vivo anti-inflammatory and antioxidant activities of Ugonin M, which is involved in inhibiting the NF-κB and MAPK signaling pathways, contributes to its hepatoprotective effects. In conclusion, the research data suggested that Ugonin M has considerable potential for development as a natural hepatoprotective agent for APAP-induced liver injury.
